# CMTX Disorder and CamKinase

**DOI:** 10.3389/fncel.2016.00049

**Published:** 2016-03-01

**Authors:** Frédéric Bihel, Burkhard Gess, Michel Fontés

**Affiliations:** ^1^Laboratoire d'Innovation Thérapeutique, Faculté de Pharmacie, UMR7200, Centre National de la Recherche Scientifique, Université de StrasbourgStrasbourg, France; ^2^Department of Sleep Medicine and Neuromuscular Disorders, University Hospital MuensterMuenster, Germany; ^3^Department of Neurology, Aachen RWTH University ClinicAachen, Germany; ^4^Nutrition, Obesity and Risk of Thrombosis Laboratory, UMR Institut National de la Santé et de la Recherche Médicale 1062, UMR INRA 1260, Aix Marseille UniversitéMarseille, France

**Keywords:** CamKinase, myelin sheath, neuropathies, genomic instability, drug development

Charcot-Marie-Tooth disease is a demyelinating peripheral neuropathy and is a heterogeneous inherited disorder (40 loci have been described so far) affecting peripheral nerves. Two forms, CMT1A and CMTX, account for 60 and 15% of patients, respectively, with clear familial transmission (Boerkel et al., 2002).

CMTX is caused by mutations in the GBJ1 gene encoding the synthesis of connexin 32 (Cx32, Bergoffen et al., 1993), which is a myelin protein related to PNS and CNS (Scherer et al., 1995). Cx32 is a membrane protein located in gap junctions, which forms hexameric hemichannels called connexons. However, the mechanism by which anomalies in connexin 32 affect myelination and function of PNS remains is still unclear. Here, we share our opinion that CamKinase are involved in the pathophysiology of CMTX and likely involve genomic instability caused by mutations in Gjb1.

## Connexin, CamKII, and genomic stability

Apart from its role in the synthesis of Cx32, an inappropriate expression of the gene Gjb1 has recently been associated with genomic instability during mitosis (Mones et al., 2012, www.mitocheck.org) leading to the hypothesis that connexins may be linked, directly or indirectly, to genomic stability. The literature suggests that at least one signaling pathway is associated with both phenomena—calcium homeostasis. Indeed, connexin 32 oligomerizes in Schwann cells to form hemichannels known to facilitate the diffusion of metabolites across the layers of myelin sheath. It is now established that calmodulin (CaM) can directly bind the C-terminus of Cx32 and regulate gap junction channels (Torok et al., 1997; Ahmad et al., 2001; Dodd et al., 2008; Stauch et al., 2012). In response to increased intracellular levels of calcium, CaM then binds to Cx32 channels triggering its closure and allowing the extrusion of Ca^2+^ via others channels to maintain calcium homeostasis. (Lurtz et al., 2003) In CMTX, anomalies of Cx32 alter the binding of CaM, leading to disturbance of calcium homeostasis by maintaining high intracellular calcium concentrations. (De Vuyst et al., 2006) However, as Cx32 anomalies decreases the binding of CaM, we can hypothesize an increase of “free” CaM in the presence of high concentrations of calcium leading to autophosphorylation of CaMKII upon activation by Ca^2+^ and CaM (Hell Johannes, 2014). Acting via a retrocontrol mechanism, CaMKII can then phosphorylate Cx32, leading to modulations of the gap junctional communications (Lampe and Lau, 2004; Stauch et al., 2012). Recently, we have shown that KN93 and KN-62—two inhibitors of reference of CaMKII—could restore connexin activity (Mones et al., 2014, p. 382), but as the level of phosphorylation of Cx32 has not been measured, we cannot make any conclusions about the mechanism involved in this overstimulation.

Genomic instability occurs when multipolar spindles appear due to centrosome overduplication. In 2002, Matsumoto and Maller described in Xenopus egg extracts that inactivation of CaMKII could block centrosome overduplication suggesting that calcium oscillations in the cell may be linked to centrosome duplication. (Matsumoto and Maller, 2002) Also also Xenopus egg extracts, Reber et al. confirmed this hypothesis and showed that calcium or constitutive active CaMKII promotes microtubule destabilization (Reber et al., 2008). We recently showed that CamKII activity is overactivated in cells from a mouse model of CMTX involving mutations in Gjb1 (Mones et al., 2014) and in cells from CMTX1 patients (Mones et al., 2015). These cells presented centrosome overduplication and genomic instability. Interestingly, this cellular phenotype was corrected by treatment with CamKII inhibitors both in Gjb1-mutated mouse and human patient cells (Mones et al., 2014, 2015).

These data suggest that anomalies of Cx32 due to GBJ1 mutations lead to unbalanced calcium homeostasis resulting in a genomic instability through the overactivation of CaMKII.

## Connexin and myelin

Myelin deficit is the first alteration observed in Cx32-deficient mice, supporting the link between connexin and myelin (Scherer et al., 1998). Cx32 is the most abundant connexin isoform in Schwann cells, and it oligomerizes to form gap junction channels between two cells. However, gap junctions in Schwann cells can also form intracellular channels through the layers of myelin providing a direct route for the diffusion of metabolites and second messengers from the Schwann cell to the axon. This is likely necessary for myelin formation (Ressot and Bruzzone, 2000). Our recent work with KN93 and KN62, two inhibitors of CaMKII, concur with this hypothesis because they may block the CaMKII-mediated phosphorylation of Cx32 and at least partially restore the activity of the connexon (Mones et al., 2014, 2015).

However, some facts challenge this mechanistic hypothesis. Indeed, Cx32 is the major component of liver gap junctions, and CMTX mutations of GJB1 do not result in a severe phenotype in this organ. Regarding CNS, although patients with central clinical signs have been reported (Hanemann et al., 2003), the majority of CMTX patients did not present central clinical signs. However, they did present infra-clinical and asymptomatic signs. This a major observation in inherited disorders caused by mutations in an ubiquitously expressed protein. There is usually no clear explanation.

## CamKII and myelin

Another hypothesis to explain the CMTX phenotype due to GJB1 mutations is overactivation of CaMKII. A recent work by Weggener et al. supports this hypothesis. They showed that a mouse line invalidated for CamKIIβ gene exhibits a deficit in myelination in the CNS. The authors suggested that this deficit is due to anomalies in phosphorylation of cytoskeletal proteins (Waggener et al., 2013). Earlier, Sahenk et al. reported that CMTX xenografts where Schwann cells bear mutations on Cx32 were presenting major cytoskeletal alterations (Sahenk, 1999). While the link between CaMKII, cytoskeleton anomalies and myelin deficits may be established, we do not yet know whether the CaMKII-related genomic instability observed in cell phenotypes of CMTX patients is part of the same signaling pathway leading to myelin deficit. However, this hypothesis is interesting because CMTX-induced genomic instability in the Schwann cells may affect the PNS myelination in a downstream process occurring over a long period of time before the appearance of clinical symptoms. In another form of CMT (CMT1A), infants were showed to bear the CMT1A mutation, but without clinical symptoms. They still had a reduced nerve conduction velocity indicating that a deficit in myelination is already present before the disease manifests (Garcia and Combarros, 1998).

This begs the question of calcium homeostasis and myelination. Nobbio et al. demonstrated the impact of perturbation of calcium homeostasis in a CMT1A model (Nobbio et al., 2009). This is strengthened by the report of Stevens et al. demonstrating that perturbation of calcium homeostasis inhibits differentiation of precursors of Schwann cells (Stevens and Fields, 2000). In the same report, they suggested that it involves Ca^2+^, calmodulin and calmodulin kinase.

## Demyelination and inflammation

Several mechanisms are likely co-existing between the primary causes of the disease (including gene mutation and the resulting deregulation of the downstream cascade) and the symptomatic manifestations of the disease (locomotion, fatigue, pain, etc.) In this field, Martini et al. demonstrated that murine models of demyelinating CMT present inflammation, in which both innate and adaptive immune systems would act as amplifiers of the primarily genetically caused neuropathies (for review, Martini and Willison, 2016). However, in their putative mechanism, they argued propose a yet to be identified primary activator that would lead to myelin phagocytosis by macrophages and Schwann cell dedifferentiation. The inflammatory process would be an aggravating factor of the neuropathy. Considering that inflammation is very frequently associated with degenerative disorders (Alzheimer's disease, diabetes …), it is likely that inflammation acts in clinical picture of demyelinating CMT. The question of cause or consequence of the disorder is difficult to answer. However, the assumption that anomalies in tissues structure (abnormal myelin, decompacted myelin, etc), are leading to inflammation is very likely.

Our opinion is that inherited demyelinating disorders are developmental disorders linked to anomalies of Schwann cells terminal differentiation and myelin formation. However, this putative role of inappropriate differentiation as the primary cause of inflammation is still to be proved. In addition, potential direct links between genomic instability and neuroinflammation remain still elusive, although Taupin reported an association between neuroinflammation in Alzheimer disease and genomic instability (aneuploidy) (Taupin, 2010). We did not find a report of a similar phenomenon in PNS disorders.

## CMTX and therapeutic approaches

Therapeutic efficacy requires a good understanding of the mechanisms involved in the disease. Recent works in the CMTX field show that there are many questions still pending before this genetic disease can be fully understood. Inflammation is certainly a good target that could lower disease severity including pain. Targeting upstream events linked to the primary cause of the disorder would be also a promising track to treat CMTX patients. Indeed, we have demonstrated that *in vitro* and *in vivo* abnormal phenotypes are corrected in animal models by treatment with CamKII inhibitors (Mones et al., 2014). In addition, we have demonstrated that the same anomalies are observed in cells from CMTX patients and that these anomalies are corrected using CamKII inhibitors. CaMKII is a common target to the various mechanistic hypotheses presented earlier. In order to prevent potential toxicity, development of CaMKII modulators may allow down-activation of CaMKII in the PNS without strongly altering calcium homeostasis in other cells. The CamKII inhibitors KN93 and KN62 have never been tested in humans and deserve a full optimization process to improve their efficacy toward CamKII. However, although no toxic evaluation has been reported, no adverse effect has been reported in animals treated with this molecule.

The hypothesis dealing with genomic instability and myelin deficit is also interesting in terms of therapeutic options. Indeed, several kinases are already known to be involved in genomic instability. These likely act downstream to CamKinases. Pim1 is one of these kinases that is involved in genomic instability in cancer cells (Roh et al., 2003). We have shown that Pim1 inhibitors can correct genomic instability in GBJ1-mutated cell lines, but were unable to correct Cx32 activity (Mones et al., 2014). It would be interesting to test these Pim1 inhibitors in CMTX mice to evaluate their ability to restore a normal phenotype, and validate or rule out this mechanistic hypothesis. Moreover, downstream kinases could be an additional therapeutic target.

## Author contributions

FB and MF wrote the manuscript. BG critically reviewed the manuscript. All authors read and approved the final version of the manuscript.

### Conflict of interest statement

The authors declare that the research was conducted in the absence of any commercial or financial relationships that could be construed as a potential conflict of interest.
